# Metabolic–epigenetic crosstalk in innate immune cell plasticity within the tumor microenvironment

**DOI:** 10.1038/s12276-026-01802-3

**Published:** 2026-08-06

**Authors:** Junho Noh, Jaehyun Lee, Chaelin You, Kyuho Kang

**Affiliations:** Department of Biological Sciences and Biotechnology, Chungbuk National University, Cheongju, Korea

**Keywords:** Cancer metabolism, Cancer microenvironment

## Abstract

The tumor microenvironment exerts profound metabolic and epigenetic pressures that shape the plasticity of innate immune cells, influencing their capacity to promote or suppress tumor progression. Emerging evidence highlights the intricate interplay between metabolic reprogramming and epigenetic modifications in macrophages, neutrophils, and other innate immune populations within the tumor microenvironment. Tumor-derived metabolites, hypoxia, and nutrient availability dynamically regulate chromatin accessibility, histone modifications, and DNA methylation patterns, thereby driving context-dependent immune phenotypes. Notably, metabolic rewiring can imprint long-lasting epigenetic changes, a phenomenon known as innate immune memory, which alters subsequent immune responses. Here, we discuss how key metabolic pathways, including glycolysis, fatty acid oxidation, and amino acid metabolism, govern innate immune cell fate and function via epigenetic mechanisms. We also highlight recent advances in epigenomic profiling that have unveiled distinct chromatin landscapes associated with innate immune dysfunction across cancer types. Finally, we explore emerging therapeutic strategies that target the metabolic–epigenetic axis to restore innate immune surveillance and enhance immunotherapy efficacy. A deeper understanding of this metabolic–epigenetic crosstalk could reveal novel avenues for modulating innate immunity in cancer therapy.

## Metabolic–epigenetic interplay shapes innate immune responses

Cancer is increasingly viewed as a dynamic ecosystem shaped by interactions between malignant cells and the tumor microenvironment. This Review examines how metabolic and epigenetic pressures within the tumor microenvironment regulate the plasticity of innate immune cells, including macrophages, neutrophils, and other innate immune populations. Tumor-derived metabolites, hypoxia, and nutrient availability modulate chromatin accessibility, histone modifications, and DNA methylation, thereby driving context-dependent immune phenotypes that either support or suppress tumor progression. The authors discuss how glycolysis, fatty acid oxidation, and amino acid metabolism govern innate immune cell fate through epigenetic mechanisms, including long-lasting imprints associated with innate immune memory. Recent epigenomic profiling is also highlighted for revealing chromatin landscapes linked to innate immune dysfunction across cancer types, supporting therapeutic targeting of the metabolic–epigenetic axis in cancer immunotherapy. This summary was initially drafted using artificial intelligence, then revised and fact-checked by the authors.

## Introduction

**Fig. 1:**
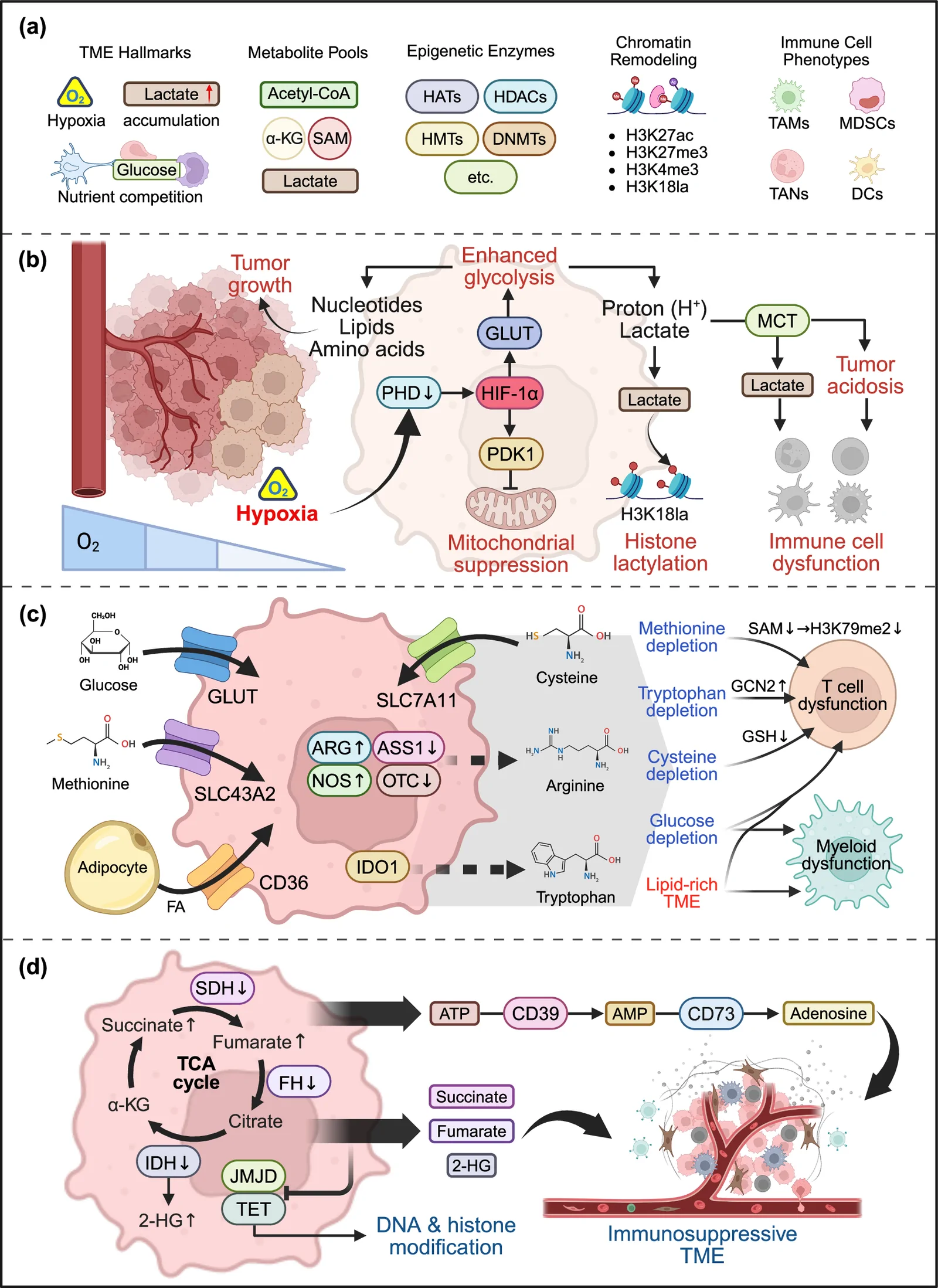
Metabolic and epigenetic determinants of innate immune cell dysfunction within the tumor microenvironment. **a** The tumor microenvironment (TME) is defined by metabolic hallmarks including hypoxia and nutrient competition. These factors reshape intracellular metabolite pools (acetyl-coenzyme A (CoA), α-ketoglutarate (α-KG), *S*-adenosylmethionine (SAM), and lactate), providing essential cofactors for chromatin-modifying enzymes (histone acetyltransferases (HATs), histone deacetylases (HDACs), histone methyltransferases (HMTs), and DNA methyltransferases (DNMTs)) and driving the epigenetic remodeling that governs the plasticity of innate immune cells (tumor-associated macrophages (TAMs), tumor-associated neutrophils (TANs), dendritic cells (DCs), and myeloid-derived suppressor cells (MDSCs)). **b** Hypoxia stabilizes HIF-1α, which shifts cancer cell metabolism toward enhanced glycolysis and mitochondrial suppression, leading to lactate export. Excess extracellular lactate induces tumor acidosis and promotes histone lactylation (H3K18la), impairing the metabolic fitness and effector function of infiltrating immune cells. **c** Tumor cells competitively deplete glucose and amino acids (methionine, tryptophan, arginine, and cystine). This nutrient scarcity activates stress pathways (for example, GCN2) and depletes SAM levels (reducing H3K79me2), while a lipid-rich environment further exacerbates T cell and myeloid cell dysfunction. **d** Dysregulated TCA cycle activity accumulates oncometabolites (succinate, fumarate, and 2-hydroxyglutarate (2-HG)) that inhibit α-KG-dependent dioxygenases (TETs and JMJDs), altering DNA/histone methylation. Concurrently, CD39/CD73-mediated generation of adenosine, an immunosuppressive purine metabolite, reinforces the tolerogenic TME. Created with BioRender.com (https://BioRender.com/3qcmwos). ARG1 arginase 1, ASS argininosuccinate synthetase, FA fatty acid, GCN2 general control non-derepressible 2, HIF hypoxia-inducible factor, IDO1 indoleamine 2,3-dioxygenase 1, MCT monocarboxylate transporter, NOS nitric oxide synthase, OTC ornithine transcarbamylase, PDK1 pyruvate dehydrogenase kinase 1, PHD prolyl hydroxylase domain, SDH succinate dehydrogenase, TCA tricarboxylic acid.

The prevailing understanding of cancer has shifted from focussing solely on the genetic alterations of malignant cells to viewing it as a complex, evolving ecosystem shaped by the tumor microenvironment (TME). This intricate milieu comprises cancer cells interwoven with a multitude of non-malignant components, including diverse immune and stromal cells (such as cancer-associated fibroblasts, endothelial cells, and pericytes), embedded within a remodeled extracellular matrix^1^. Initially viewed as passive bystanders, these host cells are now known to be actively co-opted and reprogrammed by the evolving tumor to create a tumor-supportive niche^2^. This co-option relies on a continuous and reciprocal communication within the TME, mediated primarily by paracrine signaling (cytokines, chemokines, and growth factors) and the release of extracellular vesicles^3^, and metabolic alterations^4^. This intercellular dialog drives a shift from a protective, homeostatic tissue environment toward chronic inflammation and immune suppression, which are further reinforced by hypoxia and altered nutrient availability characteristic of the TME^5–7^. Recent advances in single-cell and spatial transcriptomic analyses have provided detailed insights into the diversity and complexity of intercellular signaling networks that control TME complexity^8,9^, highlighting the importance of the TME as a therapeutic target. Figure 1a outlines the conceptual framework of this Review, in which hallmark features of the TME converge on metabolic and epigenetic programs that remodel chromatin and shape the phenotypic and functional states of innate immune cells.

Innate immune cells are central to the dysfunctional state of the TME, displaying remarkable and often paradoxical plasticity with dual roles in cancer progression^6^. Key subsets including macrophages, neutrophils, and dendritic cells (DCs) can adopt either antitumor or pro-tumor phenotypes. In their cytotoxic, pro-inflammatory states, such as M1-like macrophages and N1 neutrophils, these cells mediate tumor surveillance and T cell priming. Conversely, the immunosuppressive TME reprograms them toward pro-tumor states, including M2-like macrophages, N2 neutrophils, and myeloid-derived suppressor cells (MDSCs), which promote angiogenesis, matrix remodeling, and immune evasion via IL‑10, transforming growth factor (TGF)‑β, arginase‑1 (ARG1), and PD‑L1 (refs. ^10–12^). Their functional balance is further shaped by metabolic and epigenetic remodeling within the tumor milieu. Immune cells dynamically reprogram their metabolism, shifting between glycolysis, oxidative phosphorylation (OXPHOS), and fatty acid oxidation (FAO) in response to nutrient levels, hypoxia, and environmental cues^13,14^. These metabolic states are tightly linked to the epigenome, as metabolites such as acetyl-CoA, α-ketoglutarate (α-KG), and *S*-adenosylmethionine (SAM) act as key cofactors for chromatin-modifying enzymes^15,16^. Within the TME, metabolic stress from hypoxia, nutrient deprivation, and tumor-derived metabolites reshapes both metabolism and the epigenetic landscape of innate immune cells, thereby dictating their activation, polarization, and longevity^15,16^. This metabolic–epigenetic crosstalk underlies immune cell plasticity and contributes to phenomena such as trained immunity or immune exhaustion^16,17^. Because aberrant epigenetic states within the TME can suppress antitumor responses and limit immunotherapy efficacy, targeting this axis has emerged as a promising therapeutic strategy^18^. Here, we first outline the metabolic landscape of the TME and the distinct metabolic profiles of key innate immune subsets. We then dissect the specific metabolic–epigenetic axes, including the emerging role of histone lactylation, that drive these phenotypes. Finally, we explore the concept of innate immune memory (trained immunity) within the tumor context and discuss therapeutic strategies to target this crosstalk for cancer treatment.

## The unique metabolic landscape of the tumor microenvironment

The TME constitutes a unique and dynamically regulated metabolic ecosystem, driven by uncontrolled cancer cell proliferation, aberrant vasculature, and multifaceted metabolic and immunological stressors^19^. This niche imposes severe constraints on infiltrating immune cells by depleting essential nutrients and oxygen, while accumulating immunosuppressive metabolic by-products^20^. Understanding this foundational metabolic landscape is crucial, as it provides the environmental context for the subsequent metabolic and epigenetic reprogramming of innate immune cells. The TME metabolic landscape is characterized by four interconnected features that profoundly influence antitumor immunity: hypoxia and enhanced aerobic glycolysis, lactate accumulation and acidosis, nutrient depletion and competition, and oncometabolites and purinergic stress.

### Hypoxia and enhanced glycolysis

The TME is characterized by a unique metabolic landscape driven chiefly by hypoxia and altered cancer cell metabolism. Rapid tumor cell proliferation combined with structurally and functionally inadequate tumor vasculature causes steep oxygen gradients leading to hypoxia^21–24^. This oxygen deprivation promotes stabilization of hypoxia-inducible factors (HIFs), particularly HIF-1α, through suppression of prolyl hydroxylase domain enzymes (PHDs), as well as activation by oncogenic pathways^25–27^ (Fig. 1b).

HIF-1α acts as a master regulator of gene expression, increasing glucose transporter levels and glycolytic enzyme activity, thereby enhancing glycolysis even in the presence of oxygen, a metabolic adaptation known as the Warburg effect^25,28,29^. This metabolic rewiring favors biosynthetic precursor production (nucleotides, lipids, and amino acids) over maximal ATP generation, supporting rapid tumor growth^30^. In addition, HIF-1α induces pyruvate dehydrogenase kinase 1 (PDK1), which inhibits pyruvate entry into the tricarboxylic acid (TCA) cycle, thereby suppressing mitochondrial OXPHOS, reducing oxygen consumption and reactive oxygen species (ROS) generation^21,31^. This metabolic shift promotes the excessive production of lactate and protons (H^+^), contributing to tumor acidosis^32^ (Fig. 1b).

### Lactate accumulation and acidosis

Enhanced glycolysis in tumor cells leads to increased production of lactate and protons (H^+^), which are co-exported via monocarboxylate transporters (MCTs)^32^. This process results in the development of an acidic extracellular environment, which is associated with tumor progression and suppression of antitumor immune responses^32,33^. Elevated extracellular levels of lactate and H^+^ impair immune cell metabolism and effector function within the TME. In myeloid cells, lactate reduces glycolytic activity and tumor necrosis factor secretion in monocytes and promotes polarization of tumor-associated macrophages (TAMs) toward an M2-like, immunosuppressive state, facilitating immune evasion^34,35^. Within the lymphoid compartment, lactate impairs T cell proliferative capacity and cytokine production^36^, whereas in natural killer (NK) cells, LDHA-dependent lactic acid accumulation suppresses interferon (IFN)-γ production, partly through inhibition of NFAT activation, limiting innate antitumor surveillance^37^. Extracellular acidosis represents an additional immunosuppressive factor that impairs T cell proliferation and effector function and promotes T cell anergy, contributing to immune dysfunction in the TME^38^.

Beyond its direct metabolic effects, lactate can also function as a signaling metabolite linking cellular metabolic state to epigenetic regulation. Lactate serves as a substrate for histone lysine lactylation (Kla), a chromatin modification that connects metabolic states to transcriptional regulation^39^. In M1-polarized macrophages, histone lactylation accumulates during the later stages of activation and promotes the expression of genes associated with tissue repair and homeostasis, indicating that lactate-driven epigenetic modifications can reshape innate immune cell responses^39^. Emerging evidence suggests that sustained histone lactylation contributes to long-term epigenetic remodeling and is implicated in trained immunity^40^.

### Nutrient depletion and competition

The TME is a metabolically challenging niche characterized by profound nutrient depletion resulting from tumor cell metabolic rewiring^19^. This dysregulated nutrient landscape creates intense competition between tumor and immune cells, substantially limiting the metabolic fitness and effector functions of infiltrating immune cells^41^. Tumor cells exhibit intensified aerobic glycolysis, consuming glucose at elevated rates, and show a marked addiction to glutamine to sustain energy production and to support TCA cycle anaplerosis^42^. This heightened glutamine consumption depletes extracellular glutamine pools, constraining cytotoxic T cell activity and promoting immunosuppressive functions in myeloid cells^43,44^.

Key essential amino acids such as arginine, tryptophan, methionine, and cystine are frequently depleted within the TME^19,45^ (Fig. 1c). Arginine depletion is mediated by enhanced activity of nitric oxide synthase and arginase, compounded by reduced expression of urea cycle enzymes such as argininosuccinate synthetase 1 and ornithine transcarbamylase, rendering many tumors auxotrophic^46,47^. Tryptophan catabolism via the kynurenine pathway, chiefly regulated by indoleamine 2,3-dioxygenase 1 (IDO1), constitutes a critical immunosuppressive mechanism^48^. Tryptophan scarcity activates the general control non-derepressible 2 stress response in immune cells, leading to T cell proliferation arrest, apoptosis, and anergy^49–51^. Methionine depletion, driven by cancer cell upregulation of the transporter SLC43A2, reduces intracellular SAM, impairing methyltransferase activity and decreasing the epigenetic marker H3K79 dimethylation (H3K79me2), thereby inducing T cell functional exhaustion^52,53^. Cysteine availability, vital for maintaining cellular redox homeostasis, is compromised by cancer cells exploiting the cystine/glutamate antiporter system (xCT; SLC7A11), which sequesters cystine^54,55^. This deprives infiltrating T cells of precursors for glutathione synthesis, triggering CD36-mediated ferroptosis and dysfunction of tumor-infiltrating CD8^+^ T cells^56^.

Lipid metabolism is profoundly reprogrammed in tumors to support proliferation and survival^57^. Cancer cells predominantly rely on de novo lipogenesis, FAO, and fatty acid uptake via membrane transporters such as CD36 (refs. ^58,59^). Interactions with adipocytes facilitate tumor progression by enabling cancer cells to induce adipocyte lipolysis, thereby accessing and storing adipocyte-derived fatty acids^60,61^. The lipid-rich TME also modulates infiltrating immune cell function, fostering an immunosuppressive milieu^62,63^.

### Oncometabolites and purinergic stress

Metabolic reprogramming in the TME is characterized by accumulation of intermediate metabolites that directly modify epigenetic landscapes^64^. Loss-of-function mutations in critical TCA cycle enzymes such as succinate dehydrogenase (SDH) and fumarate hydratase (FH) cause accumulation of succinate and fumarate, respectively^65^ (Fig. 1d). These oncometabolites act as competitive inhibitors of α-KG-dependent dioxygenases, including TET DNA demethylases and Jumonji histone demethylases, leading to widespread DNA hypermethylation and transcriptional reprogramming^66,67^. Beyond epigenetic regulation, succinate and fumarate also modulate immunosuppressive functions in the TME by influencing innate immune cell polarization and cytokine production^68,69^. Recent studies have further revealed that oncometabolites accumulated within tumors are not merely metabolic intermediates but serve as direct regulators of epigenetic modifications. Notably, 2-hydroxyglutarate, produced in IDH-mutant cancers, inhibits α-KG-dependent enzymes, inducing DNA and histone methylation changes that impact not only cancer cell transcriptional programs but also the immune microenvironment^70^. Furthermore, succinate acts through its receptor SUCNR1 to modulate macrophage polarization and inflammatory responses, illustrating a paracrine mechanism by which oncometabolites influence tumor-infiltrating immune cells^68^. The TME is additionally marked by extracellular accumulation of adenosine, a potent immunosuppressive purinergic metabolite^71^. Adenosine is generated via ATP degradation catalyzed by ectonucleotidases CD39 and CD73 (ref. ^69^). High levels of adenosine engage receptors such as A2A on infiltrating immune cells, robustly suppressing antitumor immunity^69^. This purinergic stress exacerbates immune evasion by dampening T cell activation and effector responses.

## Metabolic reprogramming of innate immune cells in the TME

The metabolic landscape described earlier does not affect all immune cells uniformly as it is characterized by distinct features such as hypoxia, lactate accumulation, nutrient depletion, and oncometabolite stress. Rather, each innate immune subset interprets these environmental constraints through its own metabolic machinery, giving rise to divergent functional states. Metabolic reprogramming of innate immune cells has emerged as a defining hallmark of the TME. By adapting to nutrient deprivation, hypoxia, and tumor-derived metabolites, these cells rewire their metabolic pathways to maintain viability and acquire specialized functions that can either promote or restrict tumor progression^6^. In the following section, we discuss the distinct metabolic profiles of major innate immune subsets, highlighting how nutrient availability and metabolic shifts determine their functional plasticity within the TME. Although T cell subsets are also extensively regulated by metabolic and epigenetic mechanisms within the TME, this Review focusses on innate immune cell-intrinsic pathways while discussing their consequences for T cell responses where relevant.

### Tumor-associated macrophages

**Fig. 2:**
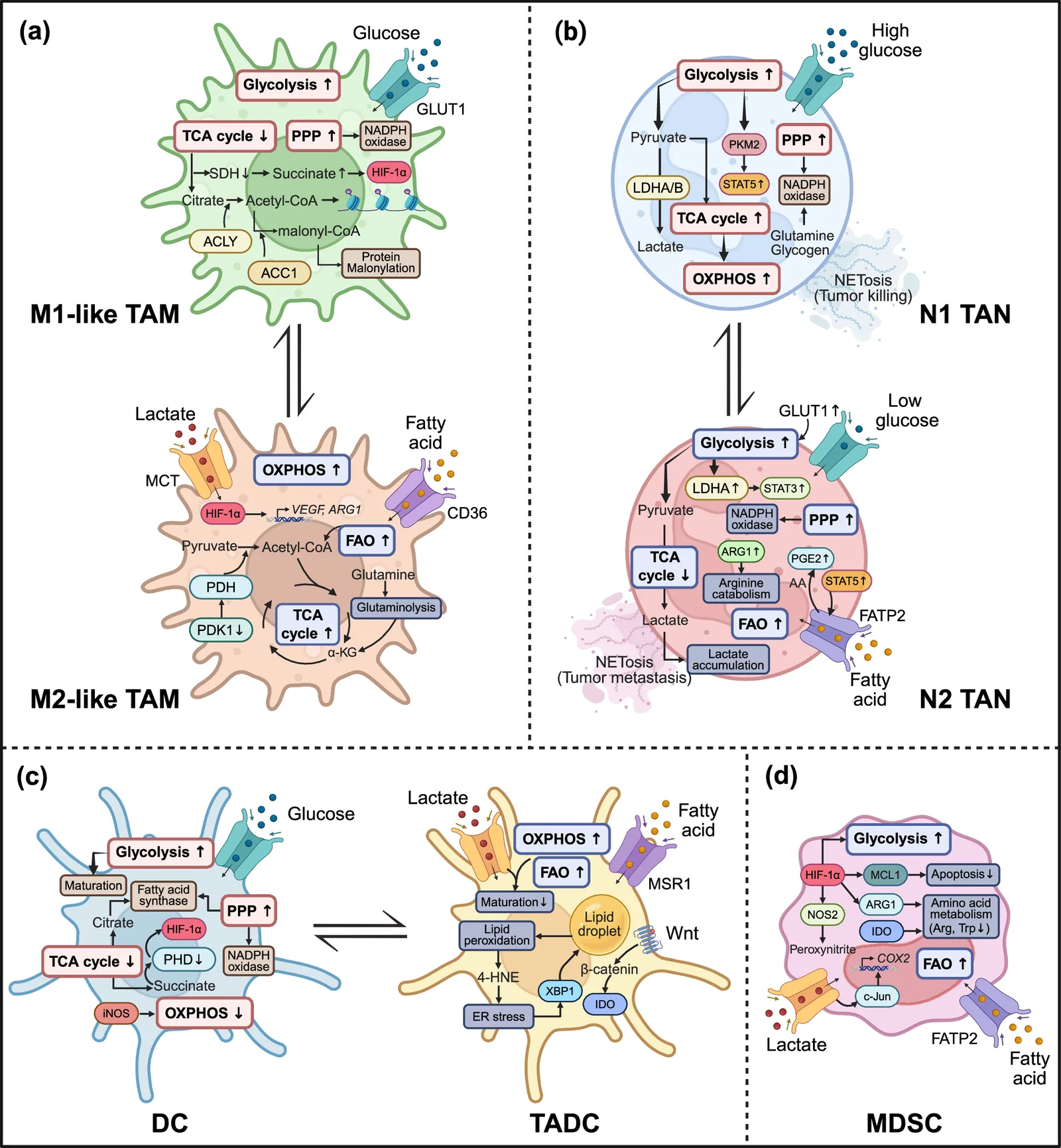
Divergent metabolic programs orchestrate the dichotomy between antitumor and pro-tumor phenotypes in myeloid cells. **a** M1-like tumor-associated macrophages (TAMs) utilize aerobic glycolysis and the pentose phosphate pathway (PPP) for inflammation, with ATP-citrate lyase (ACLY)-derived acetyl-coenzyme A (CoA) supporting fatty acid synthesis and protein malonylation while succinate stabilizes HIF-1α. Conversely, M2-like TAMs rely on CD36-mediated lipid uptake, fatty acid oxidation (FAO), and oxidative phosphorylation (OXPHOS). Reduced PDK1 promotes pyruvate oxidation and glutaminolysis replenishes the TCA cycle, whereas tumor-derived lactate induces *VEGF* and *ARG1* via HIF-1α. **b** Under high glucose, N1 tumor-associated neutrophils (TANs) increase glycolysis and TCA activity via the PKM2–STAT5 axis, whereas the PPP, glutamine, and glycogen collectively drive NADPH oxidase-mediated reactive oxygen species for tumor-killing NETosis. By contrast, N2 TANs adapt to nutrient-deprived niches by upregulating GLUT1 and LDHA-mediated lactate for STAT3 activation. STAT5-induced FATP2 promotes arachidonic acid (AA)-mediated prostaglandin E2 (PGE2) synthesis, and a dysfunctional TCA cycle alongside high ARG1 triggers metastatic NETosis. **c** Immunogenic dendritic cells (DCs) mature via glycolysis and utilize the PPP for fatty acid synthesis and NADPH oxidase activation. A truncated TCA cycle stabilizes HIF-1α via succinate, whereas iNOS-derived NO suppresses OXPHOS. Conversely, tumor-associated DCs (TADCs) exhibit increased lactate uptake, FAO, and OXPHOS to hinder maturation. Upregulated MSR1 drives lipid droplet formation and peroxidation, triggering XBP1-mediated ER stress and impairing antigen presentation, while Wnt signaling promotes tolerance via β-catenin and indoleamine 2,3-dioxygenase 1 (IDO1). **d** Myeloid-derived suppressor cells (MDSCs) activate HIF-1α to maintain glycolysis, while MCL1 limits their apoptosis. In parallel, ARG1 and IDO1 facilitate amino acid depletion. Furthermore, NOS2-derived peroxynitrite and the uptake of lactate and fatty acids induce *COX2* and FAO to sustain immunosuppressive functions. Created with BioRender.com (https://BioRender.com/l1dojjv). ARG arginase, ER endoplasmic reticulum, HIF hypoxia-inducible factor, IDO1 indoleamine 2,3-dioxygenase 1, iNOS inducible nitric oxide synthase, MCT monocarboxylate transporter, PDK1, pyruvate dehydrogenase kinase 1, PHD prolyl hydroxylase domain, SDH succinate dehydrogenase, TCA tricarboxylic acid, α-KG α-ketoglutarate.

TAMs demonstrate exceptional metabolic plasticity, with their functional phenotypes existing along a continuous spectrum rather than within rigid classes^72^. This metabolic flexibility allows them to adapt to the TME’s unique stressors, where specific metabolic shifts dictate whether they promote or restrict tumor progression (Fig. 2a).

#### M1-like TAMs: metabolic rewiring for pro-inflammatory effector functions

Antitumor M1-like macrophages are characterized by an early and robust increase in both aerobic glycolysis and mitochondrial respiration, supported by elevated glucose uptake^73,74^. This glycolytic flux further feeds into the pentose phosphate pathway (PPP) to generate the NADPH necessary for ROS production^75^. A key feature of this state is the disrupted TCA cycle, in which the downregulation of enzymes such as IDH and SDH leads to the intracellular accumulation of citrate and succinate^76^. Citrate exported from the mitochondria is converted by ACLY into acetyl-CoA, which serves as a dual-purpose substrate for histone acetylation at inflammatory gene loci and for de novo fatty acid synthesis (FAS)^77^. Through the activity of ACC1, acetyl-CoA is further processed into malonyl-CoA, which promotes pro-inflammatory gene expression via protein malonylation^78^. Crucially, the accumulated succinate provides a vital link to functional acquisition by acting as a competitive inhibitor of prolyl hydroxylase, thereby stabilizing HIF-1α and amplifying inflammatory signaling^79^. This process is accompanied by mitochondrial reverse electron transport, which generates additional ROS to enhance antimicrobial and tumor-killing functions^79^. Beyond their direct tumoricidal activity, M1-like TAMs serve as critical orchestrators of adaptive immunity. Notably, they can cross-present antigens to prime naive CD8^+^ T cells and activate memory CD8^+^ T cells^80^. Furthermore, the secretion of IL-12 and IL-18 by M1-like cells promotes the differentiation of T_H_1 effector cells and enhances the cytotoxic potential of CD8^+^ T cells, effectively countering the metabolic and signaling cues that drive T cell exhaustion^81^. Additionally, metabolism-driven production of T_H_1-recruiting chemokines, such as CXCL9 and CXCL10, ensures the infiltration of effector T populations into the TME^82^, thereby transforming a cold tumor into an immunologically active hot environment. Ultimately, these metabolic adaptations enable M1-like TAMs to effectively prime T cells and drive potent antitumor T_H_1 responses.

#### M2-like TAMs: oxidative persistence and immunosuppressive dominance

In contrast to the transient inflammatory burst of M1-like cells, pro-tumor M2-like TAMs primarily rely on OXPHOS and FAO to sustain long-term immunosuppression^73,74^. This metabolic profile is established through specific enzymatic switches, such as the inhibition of PDK1, which promotes pyruvate conversion to acetyl-CoA via the PDH complex to fuel mitochondrial respiration^83^. To support this oxidative program, M2-like TAMs upregulate scavenger receptors such as CD36 for increased fatty acid uptake from the TME. Furthermore, they engage in high rates of glutaminolysis to generate α-KG, which acts as a critical anaplerotic substrate to replenish the TCA cycle and sustain the bioenergetic demands of constant cytokine production^84,85^. The acquisition of these metabolic functions directly dictates the exclusion and dysfunction of infiltrating T cells through both nutrient competition and the accumulation of suppressive by-products. A hallmark of this interaction is the sensing of tumor-derived lactate, which is taken up via MCTs to stabilize HIF-1α, subsequently inducing high expression of *VEGF* and *ARG1* (refs. ^35,86^). Crucially, the resulting depletion of extracellular L-arginine by ARG1 acts as a potent metabolic stressor that impairs TCR signaling in effector T cells, driving them toward functional exhaustion or proliferative arrest^87^. Furthermore, the lipid-driven metabolism of M2-like TAMs facilitates the secretion of immunosuppressive mediators such as IL-10 and TGF-β, which not only dampen the cytotoxic potential of CD8^+^ T cells but also actively promote the recruitment and metabolic stability of regulatory T cells^88^. By coupling nutrient deprivation with suppressive signaling, M2-like TAMs reinforce a tolerogenic niche within the TME.

### Tumor-associated neutrophils (TANs)

TANs are metabolically flexible immune cells that display divergent phenotypes with distinct functional roles within the TME^12^ (Fig. 2b). Unlike TAMs, which exhibit clear metabolic polarization, both N1 and N2 TANs rely heavily on glycolysis but differ markedly in their lipid utilization and mitochondrial plasticity. This metabolic adaptability allows TANs to dictate the immune tone of the TME by either supporting or suppressing adaptive T cell responses^89^.

#### N1 TANs: glycolytic activation and effector T cell support

The antitumor N1 phenotype, induced by IFN-β, maintains a balanced metabolic state with robust glycolytic activity. This state is characterized by rapid energy production and the generation of ROS, nitric oxide (NO), and pro-inflammatory cytokines such as tumor necrosis factor-α, which enable direct tumor cell killing^89^. In this context, the PPP provides the essential NADPH required for ROS production and neutrophil extracellular trap (NET) formation, a process sustained even under nutrient-limited conditions through the metabolic flexibility of N1 TANs in utilizing internal glycogen stores and exogenous glutamine as compensatory substrates to maintain NADPH oxidase activation^90,91^. This functional acquisition is further supported by enhanced glycolytic flux, which activates the PKM2–STAT5 axis to sustain the effector potential of the TME^90,92^. Additionally, the conversion of pyruvate into lactate by LDHA and LDHB facilitates lactate-driven NETosis, which enhances the innate surveillance capacity of N1 TANs by physically entrapping and neutralizing malignant cells^90,93^. Beyond direct tumoricidal activity, N1 TANs orchestrate adaptive immunity by exhibiting antigen-presenting cell-like capacities^94^. They upregulate HLA-DR and co-stimulatory molecules such as CD86 and 4-1BBL, initiating a positive feedback loop of T cell proliferation and IFN-γ release^94,95^. Concurrently, N1 TANs modulate various T cell subsets by enhancing the cytotoxicity of effector populations while selectively suppressing pro-tumorigenic IL-17-producing T cells through ROS-mediated mechanisms^96^. By also promoting macrophage-derived IL-12, N1 TANs foster a robust, immunologically active microenvironment that supports sustained antitumor immune surveillance^97^.

#### N2 TANs: metabolic adaptation and T cell suppression

By contrast, the pro-tumor N2-like phenotype, driven by TGF-β and granulocyte colony-stimulating factor, adapts to the hypoxic and nutrient-deprived TME by upregulating both glycolysis and FAO^89^. This metabolic shift is achieved through the increased expression of the glucose transporter GLUT1 and the fatty acid transporter FATP2 (ref. ^90^). Unlike N1-like cells, N2-like TANs exhibit reduced TCA cycle activity, favoring a metabolic shift toward pyruvate-to-lactate conversion^89,98^. Notably, although N1-derived NETs contribute to tumor surveillance, the NETosis frequently observed in N2 populations acts as a critical mechanism for remodeling the TME to facilitate cancer metastasis^99,100^. Furthermore, metabolites such as 27-hydroxycholesterol (27-HC) facilitate the recruitment of these suppressive N2 TANs to promote angiogenesis and tumor progression^101^. Together, these metabolic adaptations reinforce an immunosuppressive state that impairs T cell activity through multiple mechanisms. For instance, the elevated expression of ARG1 enhances arginine catabolism, leading to rapid arginine depletion in the TME that suppresses arginine-dependent T cell activation^102^. Additionally, STAT5-driven upregulation of FATP2 facilitates the uptake of arachidonic acid and the synthesis of prostaglandin E2 (PGE2), which further reinforces the immunosuppressive environment^103^. In specific contexts such as hepatocellular carcinoma (HCC), serum amyloid A stimulates glycolysis and increases LDHA expression, which in turn activates STAT3 signaling to upregulate PD-L1 (ref. ^104^). In this context, metabolically induced PD-L1 expression probably contributes to T cell dysfunction and tumor progression.

### Dendritic cells and myeloid-derived suppressor cells

#### DCs: metabolic maturation versus TME-induced dysfunction

DCs are critical innate immune effectors that bridge innate and adaptive immunity, yet they face substantial metabolic challenges within the TME (Fig. 2c). Upon activation, DCs typically undergo a profound metabolic switch from OXPHOS to aerobic glycolysis, a transition essential for moving from an immature tolerogenic state to a mature immunostimulatory phenotype^105,106^. During this maturation, the PPP is upregulated to produce NADPH, which supports the respiratory burst and fuels the FAS required for the expansion of the endoplasmic reticulum (ER) and Golgi apparatus, a structural remodeling that is essential for the increased cytokine secretion and antigen presentation needed to prime naive T cells^106,107^. Mechanistically, a truncated TCA cycle leads to the accumulation of citrate and succinate. Although citrate fuels FAS, succinate resulting from the suppression of SDH inhibits PHDs to stabilize HIF-1α, thereby directly promoting the expression of pro-inflammatory cytokines including IL-1β and IL-12 (ref. ^108^). At the same time, iNOS-derived NO inhibits mitochondrial electron transport to suppress OXPHOS, which reinforces glycolytic dependency and provides the necessary bioenergetic support for major histocompatibility complex expression and effector T cell activation^109,110^.

By contrast, tumor-associated DCs (TADCs) within the nutrient-deprived TME often fail to achieve this mature state owing to a forced reliance on FAO and OXPHOS^111^. Although FAO supports the energy needs of quiescent or regulatory DCs, chronic exposure to tumor-derived lipids induces the upregulation of scavenger receptors such as MSR1 and CD36, leading to aberrant lipid droplet accumulation and subsequent lipid peroxidation^112^. This oxidative process generates toxic by-products such as 4-HNE, which triggers sustained ER stress via the XBP1 axis to impair major histocompatibility complex class I synthesis and antigen cross-presentation^113^. Consequently, these lipid-laden DCs exhibit a fundamental defect in their capacity to process exogenous antigens and fail to effectively trigger antitumor T cell responses, which ultimately fosters a state of immune tolerance within the TME^112^. This suppressive environment is further reinforced by high levels of tumor-derived lactate, which actively blocks the glycolytic flux essential for immunostimulatory maturation^114^. Parallel to this metabolic inhibition, signaling through the WNT5A–β-catenin axis reprograms the secretory profile of TADCs by inducing the production of IDO1 and anti-inflammatory mediators such as IL-10 and TGF-β^111,115^. This functional transition culminates in the increased surface expression of inhibitory molecules such as *CD274*, enabling TADCs to directly promote T cell exhaustion through checkpoint signaling^115^. Collectively, these coordinated metabolic and molecular shifts prevent the generation of robust effector T cell responses and instead drive the expansion of regulatory T cells to effectively silence the initiation of adaptive antitumor immunity^116^.

#### MDSCs: metabolic plasticity and acquisition of suppressive function

MDSCs represent a heterogeneous population of immature myeloid cells that exploit metabolic plasticity to suppress antitumor immunity and promote tumor progression within the TME (Fig. 2d). Within this niche, tumor-derived lactate serves as a critical signal that diverts myeloid differentiation toward a suppressive fate, where its uptake via MCT2 stabilizes c-Jun by preventing FBW7-mediated degradation and activates *COX2,* diverting M-MDSC differentiation toward the granulocytic MDSC fate^117^. Once established, the persistence and bioenergetic requirements of MDSCs are primarily governed by the hypoxic stabilization of HIF-1α (ref. ^118^). By accelerating glycolytic flux, HIF-1α supports MDSC survival in the nutrient-deprived TME, whereas the anti-apoptotic protein MCL1 limits their apoptosis. HIF-1α-induced miR-210 further augments arginase activity and NO production^119^. To further fuel their energy-demanding suppressive activities, MDSCs upregulate scavenger receptors such as CD36 and FATP2 to enhance fatty acid uptake, which drives mitochondrial FAO and maintains high ATP levels^120^. These metabolic adaptations collectively provide the functional basis for the diverse immunosuppressive strategies that MDSCs deploy to neutralize T cell responses. Specifically, the induction of NOS2 and ARG1 by HIF-1α enables the production of high levels of ROS and reactive nitrogen species, such as peroxynitrite, which mediate the nitration of T cell receptors to disrupt antigen recognition^118,121^. Complementing this oxidative stress, the overexpression of ARG1 and IDO1 facilitates the depletion of extracellular L-arginine and tryptophan, which directly suppresses T cell proliferation and induces functional exhaustion^122,123^. Collectively, this coordinated metabolic reprogramming enables MDSCs to establish a broad suppressive barrier against effector cell recruitment and function.

## The core mechanism: metabolic–epigenetic crosstalk

The TME fundamentally reshapes the metabolite pools of TAMs, DCs, and MDSCs, thereby dictating their fate^124,125^. Hallmarks of the TME, such as hypoxia, nutrient competition, and metabolite accumulation, alter the availability of acetyl-CoA, SAM, NAD^+^, α-KG, succinate, and lactate^124,125^. These metabolites act as direct substrates or cofactors for epigenetic and epitranscriptomic enzymes, linking metabolic rewiring to transcriptional states and functional phenotypes^18,126^. Network-level analyses confirm that metabolic modules govern macrophage polarization^108^, and dysregulation of these axes influences responses to cancer immunotherapies^18^. The following sections focus on three core metabolic–epigenetic axes, specifically acetyl-CoA/NAD^+^, α-KG/succinate, and one-carbon/SAM, alongside the emerging role of histone lactylation.

### The acetyl-CoA/NAD^+^ axis

**Fig. 3:**
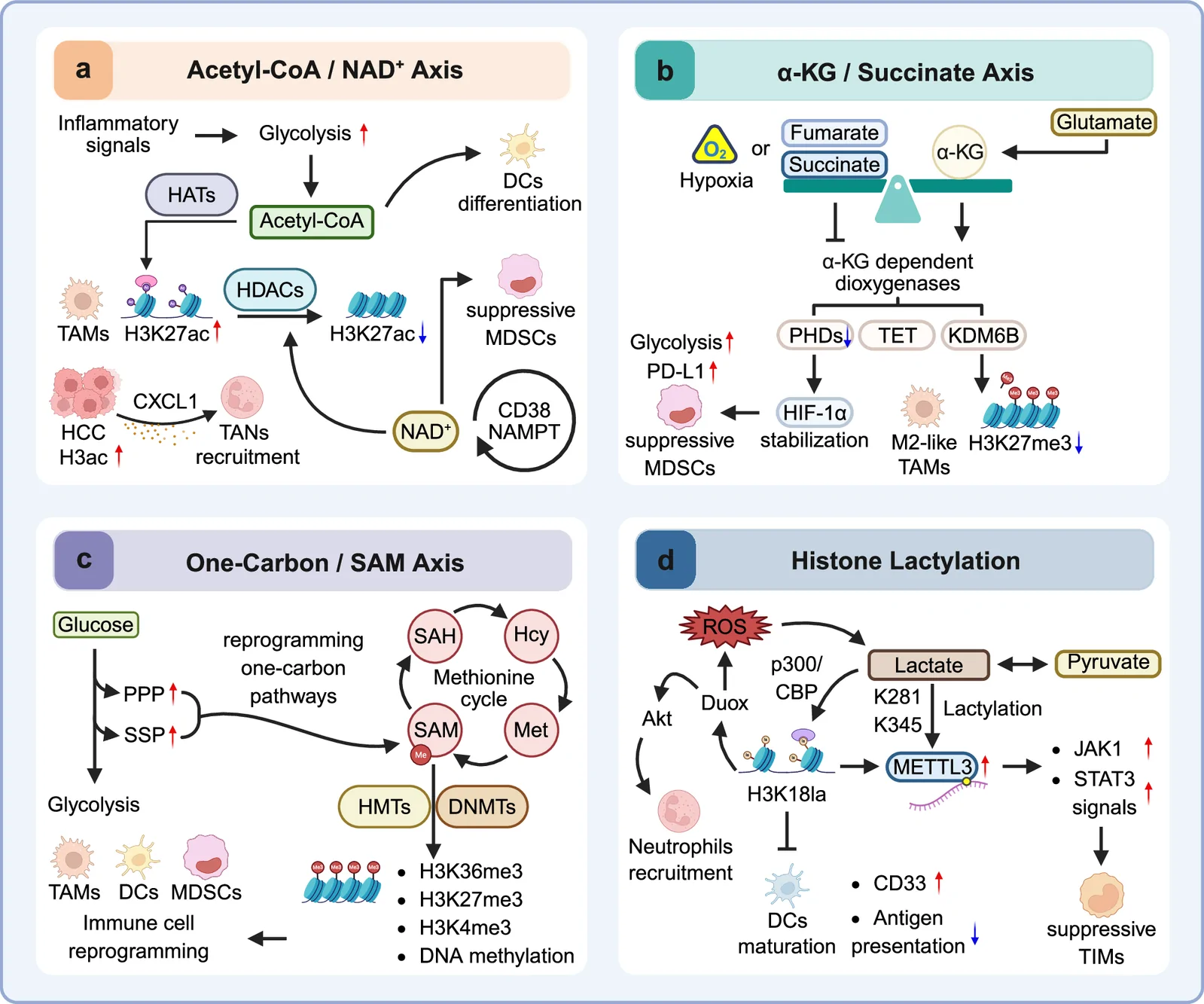
Core metabolic–epigenetic axes governing innate immune cell states in the tumor microenvironment. **a** Acetyl-coenzyme A (CoA)/NAD^+^ axis. Inflammatory signals and glycolysis-driven citrate export generate acetyl-CoA that fuels histone acetyltransferase (HAT)-mediated H3K27ac in tumor-associated macrophages (TAMs) and H3 acetylation in hepatocellular carcinoma (HCC) cells, thereby recruiting tumor-associated neutrophils (TANs) and shaping dendritic cell (DC) differentiation. In parallel, heterogeneous NAD^+^ metabolism (CD38/NAMPT) regulates Sirtuin/histone deacetylase (HDAC) activity, contributing to resolution programs and suppressive myeloid-derived suppressor cell (MDSC) phenotypes. **b** α-ketoglutarate (α-KG)/succinate axis. The balance between α-KG and succinate/fumarate regulates the activity of α-KG-dependent dioxygenases, including prolyl hydroxylase domains (PHDs), TET DNA demethylases, and KDMs. Hypoxia or succinate/fumarate accumulation inhibits these dioxygenases, particularly PHDs, thereby stabilizing hypoxia-inducible factor (HIF)-1α and promoting glycolytic reprogramming, increased PD-L1 expression, and the suppressive program of MDSCs. Conversely, glutaminolysis-derived α-KG supports KDM6B-mediated H3K27me3 demethylation, promoting M2-like TAM polarization. **c** One-carbon/*S*-adenosylmethionine (SAM) axis. Glucose metabolism feeds the SSP, pentose phosphate pathway (PPP), and methionine cycle to generate the universal methyl donor SAM. SAM supports histone methyltransferase (HMT)/DNA methyltransferase (DNMT)-mediated histone (H3K36me3 and H3K4me3) and DNA methylation, orchestrating the epigenetic reprogramming and proliferation of TAMs, DCs, and MDSCs. **d** Histone lactylation axis. Lactate promotes p300/CBP-mediated H3K18la deposition, thereby upregulating CD33 and inhibiting DC maturation. Furthermore, lactate-induced H3K18la and direct lactylation of METTL3 at K281/K345 enhance m^6^A-dependent JAK1–STAT3 signaling, establishing immunosuppressive phenotypes in tumor-infiltrating myeloid cells (TIMs). Independently, a H3K18la–DUOX–reactive oxygen species (ROS) positive-feedback loop drives excessive neutrophil recruitment in inflammatory contexts. Created with BioRender.com (https://BioRender.com/o1vjl4d).

Histone acetylation, such as H3K27ac, is a permissive mark that depends on nucleocytosolic acetyl-CoA to fuel histone acetyltransferases^126^. In macrophages, inflammatory stimuli and Toll-like receptor signaling enhance glycolysis and promote the export of mitochondrial citrate, which is then cleaved by ATP-citrate lyase (ACLY) to generate acetyl-CoA, enabling H3K27ac deposition at immune loci^77^ (Fig. 3a). In HCC models, tumor-cell acetyl-CoA accumulation increases histone H3 acetylation at the CXCL1 promoter, leading to CXCL1 secretion and recruitment of TANs that promote metastasis^127^. Acetyl-CoA also regulates DC differentiation and functional adaptability^128^. Within the TME, lactate can be converted to pyruvate and subsequently to acetyl-CoA, supporting M2-associated acetylation^129^. Conversely, the intracellular CoA pool governs both metabolic state and histone acetylation levels in inflammatory macrophages^130^. Deacetylation is mediated by histone deacetylases, including the NAD^+^-dependent Sirtuin family^131^. NAD^+^ metabolism in the TME is heterogeneous and regulated by enzymes such as CD38 and nicotinamide phosphoribosyltransferase^131^. Fluctuations in NAD^+^ availability modulate Sirtuin activity and thereby alter the epigenetic state and function of TAMs and MDSCs^131,132^. In models of acute inflammation and endotoxin tolerance, NAD^+^-dependent SIRT1 and SIRT6 coordinate a metabolic switch from glycolysis to FAO^133^, and SIRT1 deacetylates RelA/p65 and H4K16 to terminate inflammatory responses^134^. Because MDSCs proliferate rapidly, they likely exploit this NAD^+^–Sirtuin axis to maintain an immunosuppressive phenotype.

### The α-KG/succinate axis

The ratio of α-KG to succinate and fumarate acts as a rheostat for α-KG-dependent dioxygenases, such as lysine demethylases, TET DNA demethylases, and prolyl hydroxylases^135,136^ (Fig. 3b). α-KG is an essential cofactor, whereas succinate and fumarate, which accumulate under hypoxia or due to mutations in SDH or FH, competitively inhibit these enzymes^136^. In MDSCs, succinate accumulation or hypoxia inhibits PHDs and stabilizes HIF-1α (refs. ^21,136^). Stabilized HIF-1α drives glycolytic reprogramming and increases PD-L1 expression^118,137^ while inducing ENTPD2/CD39L1 to convert extracellular ATP to 5′-AMP, thereby suppressing MDSC differentiation and promoting their accumulation in the TME^138^. Tumors also secrete succinate that binds the SUCNR1 receptor on macrophages or deliver it via microparticles^68,139^. Activation of this pathway induces IL-1β and ROS and upregulates ARG1 (refs. ^68,79,140^). High ARG1 activity depletes extracellular arginine and suppresses T cell proliferation^87,141^. Conversely, glutaminolysis-derived α-KG fuels KDM6B (Jmjd3) demethylase activity, removing H3K27me3 and promoting M2 polarization^84^. Thus, α-KG sustains dioxygenase activity, whereas succinate and fumarate inhibit it, stabilizing HIF-1α .

### The one-carbon/SAM axis

One-carbon metabolism and the methionine cycle generate SAM, the universal methyl-donor substrate for histone and DNA methyltransferases^135,142^ (Fig. 3c). Pro-inflammatory (M1) macrophages redirect glucose through the serine–glycine pathway and PPP to support SAM production and maintain activating histone marks such as H3K36me3 (ref. ^142^). Within the TME, TAMs from gastric cancer upregulate methionine adenosyltransferase 2A (MAT2A), elevating SAM levels and depositing H3K4me3 at promoters such as *RIP1*, thereby driving pro-tumor gene expression^143^. Given the high proliferative rate of MDSCs and the differentiation programs of DCs, SAM availability becomes a critical determinant of their epigenetic programming and fate decisions.

### Emerging modifications and crosstalk: histone lactylation

Lactate accumulation in the TME is now recognized as a direct substrate for histone lysine lactylation^39,144^ (Fig. 3d). p300 catalyzes the transfer of lactoyl groups to histones, whereas class I HDACs (HDAC1–3) function as delactylases^39,145^. Lactate and acidosis inhibit HDAC activity and suppress T cell and NK cell functions^36,37,146^. In DCs, reduced expression of the mitochondrial pyruvate carrier (MPC) in tumor cells increases lactate production, and the resulting TME-derived lactate induces H3K18la and upregulates the immaturity marker CD33 to inhibit DC maturation and antigen presentation^147^. In tumor-infiltrating myeloid cells (TIMs) and MDSCs, lactate-induced H3K18la transcriptionally activates the m^6^A writer METTL3 and directly lactylates METTL3 protein at K281/K345, enhancing its RNA-binding capacity and promoting JAK1–STAT3 signaling^148^. In monocyte-derived macrophages in glioblastoma, high glycolytic flux and lactate accumulation drive Kla to induce IL-10 (ref. ^149^). Lactate-driven metabolic reprogramming in macrophages has been linked to increased PD-L1 expression and immunosuppressive function^150^. In parallel, lactate-derived histone lactylation, mediated by p300, promotes H3K18la accumulation and induction of reparative genes such as *ARG1*, supporting a shift toward an M2-like phenotype^39^. A positive feedback loop between H3K18la, dual oxidase (DUOX) and ROS drives excessive neutrophil recruitment^151^. Together, lactate accumulation and histone lactylation integrate metabolic waste signals with chromatin remodeling, completing the spectrum of metabolic–epigenetic axes that program innate immune cell plasticity.

## Innate immune memory (“trained immunity”) in cancer

The metabolic–epigenetic axes outlined earlier operate acutely to shape innate immune phenotypes, yet growing evidence indicates that these chromatin modifications can persist well beyond the initial stimulus. This durability suggests that the TME may imprint lasting functional states in innate immune cells through metabolic–epigenetic mechanisms. In this context, trained immunity provides a useful conceptual framework. Trained immunity refers to the capacity of innate immune cells to adopt long-lasting functional states after restimulation without antigen-specific recombination, and its consensus definition, nomenclature and scope have been consolidated^152–154^. Short-term innate immune memory can involve inducible non-coding RNAs and transient chromatin changes^155^, whereas trained immunity is maintained through durable epigenetic reprogramming.

### Core mechanism: metabolic–epigenetic imprinting

Training stimuli such as Bacillus Calmette–Guérin (BCG) or β-glucan engage the Akt/mTOR/HIF-1α pathway and enhance glycolysis, increasing the availability of acetyl-CoA and SAM for epigenetic enzymes^156,157^. This metabolic shift reprograms chromatin marks at enhancers and promoters^156^. Enhancers are characterized by H3K4me1 (priming) and H3K27ac (activation), whereas promoters carry H3K4me3, and their coordinated remodeling during monocyte-to-macrophage differentiation establishes a primed enhancer–promoter landscape that underlies trained immunity^158–160^. β-Glucan can reverse lipopolysaccharide-induced tolerance by restoring activated enhancer signatures, demonstrating that the tolerance–training axis is reversible and epigenetically controlled^161^. Akt/mTOR-mediated metabolic reprogramming also leads to increased lactate production and stabilization of HIF-1α, linking metabolite availability to histone modifications^162^.

### Beyond the periphery: HSPC-level memory

Trained immunity can be imprinted in hematopoietic stem and progenitor cells (HSPCs), resetting the long-term immune tone^163^. BCG educates HSCs to generate myeloid progeny with heightened innate protection, and β-glucan-driven reprogramming of myelopoiesis is integral to trained immunity^163,164^. Multi-omics vaccination data show that specific epigenetic cell states after BCG vaccination predict the magnitude of trained immunity^165^. Viral infection can leave epigenetic memory in innate cells and progenitors that alters subsequent responsiveness^166^. In humans, BCG vaccination imprints a persistent transcriptional myeloid bias in HSPCs and conveys epigenetically primed signatures to circulating monocytes^167^.

### Trained immunity in the tumor microenvironment: a double-edged sword and the IFN-γ switch

Within the TME, the clinical success of intravesical BCG in bladder cancer suggests that trained immunity inducers can re-train immunosuppressive TAMs and MDSCs toward antitumor phenotypes^153,158,165^. This adaptive training is governed by the IFN-γ signal: IFN-γ actively represses the M2 program by disassembling MAF-bound enhancers while potentiating core M1 programs by selectively suppressing feedback enhancers^168,169^. IFN-driven chromatin programs in the TME track with immune checkpoint blockade sensitivity or resistance, and successful anti-PD-1 therapy requires cDC1-derived IL-12 and T cell-derived IFN-γ crosstalk^170,171^. When this axis is abrogated, such as by tumor-intrinsic β-catenin signaling that prevents cDC1 recruitment, antitumor training fails^172^. Conversely, chronic stimulation by damage-associated molecular patterns or lifestyle-associated molecular patterns can epigenetically stabilize maladaptive innate immune programs^173^.

## Therapeutic strategies targeting the metabolic–epigenetic axis

As the dysfunction of innate immune cells becomes entrenched through metabolic–epigenetic crosstalk, this section discusses metabolic modulators, epigenetic drugs, and rational combination therapies targeting this regulatory axis to effectively restore immune function.

### Pharmacological modulation of metabolism

Diverse pharmacological strategies target specific metabolic nodes to restore the antitumor efficacy of innate immune populations (Table 1). Glucose metabolism represents a complex therapeutic target because global glycolytic inhibition with 2-deoxyglucose (2-DG) broadly impairs macrophage function. This lack of specificity highlights the need for selective modulation. For instance, activation of PKM2 by compounds such as DASA-58 or TEPP-46 can attenuate excessive M1-like inflammatory responses^174^. Conversely, targeting the stress-response regulator REDD1 to enhance glycolytic flux in TAMs restricts glucose availability for endothelial cells and effectively curbs vascular hyperactivation^175^. Tumor-derived lactate forms a prototypical immunosuppressive axis by stabilizing HIF-1α in TAMs, which promotes the expression of *ARG1* and *VEGF* to facilitate angiogenesis and immunoregulation. Blocking lactate transporters or HIF-1α disrupts this program and hinders tumor growth^35^. In DCs, glycolysis is indispensable for effective immune activation as treatment with the glycolytic inhibitor 2-DG markedly impairs their function^106^. STING agonists trigger LDHA-dependent glycolytic bursts that are crucial for STING phosphorylation, type I interferon production, and subsequent CD8^+^ T cell priming^176^.

**Table 1:** Metabolic modulators regulating innate immune function in the tumor microenvironment.

Drug	Category	Target cells	Key mechanisms	Core immune effect	Refs.
2-Deoxyglucose (2-DG)	Glycolytic inhibitor	Macrophages, dendritic cells (DCs)	Global glycolytic inhibition	Impairs macrophage/DC function	^106,174^
DASA-58, TEPP-46	PKM2 activator	Macrophages	PKM2 activation	Attenuates excessive M1-like inflammatory responses	^174^
Lactate transporter blockers	Lactate transport inhibitor	Macrophages	Disrupts lactate/hypoxia-inducible factor (HIF)-1α axis	Hinders tumor growth, disrupts Arg1/VEGF program	^35^
HIF-1α inhibitors	HIF-1α inhibitor	Macrophages	Disrupts lactate/HIF-1α axis	Hinders tumor growth, disrupts Arg1/VEGF program	^35^
STING agonists	STING agonist	DCs	Triggers LDHA-dependent glycolytic bursts, STING phosphorylation	Type I interferon production, CD8^+^ T cell priming	^176^
FATP2 inhibitors	Fatty acid (FA) transport inhibitor	Myeloid-derived suppressor cells (MDSCs), neutrophils	Inhibits FA transport, reduces prostaglandin E2 (PGE2) synthesis	Attenuates immunosuppressive functions, slows tumor progression	^103^
Inhibitors of S100A4–PPARγ –FA oxidation (FAO) axis	PPARγ/FAO axis inhibitor	Macrophages	Disrupts S100A4–PPARγ–FAO lipid metabolism	Reprograms tumor-associated macrophages (TAMs) toward inflammatory/antitumor states	^178^
Etomoxir	FAO inhibitor	MDSCs	Inhibits FAO, reduces oxygen consumption	Restores T cell proliferation, enhances immunotherapy efficacy	^120^
FAO inhibitors	FAO inhibitor	DCs	FAO inhibition	Enhances DC activation	^115^
Aspirin, celecoxib	COX inhibitor	DCs, MDSCs	Modulates broader lipid metabolism (COX inhibition)	Influences DC and MDSC function	^103,180,181^
A2AR inhibitors	Adenosine axis inhibitor	Macrophages, MDSCs, DCs	Blocks adenosine signaling pathway	Disrupts key immunosuppressive pathways	^182,183^
CB-1158	ARG1 inhibitor	Macrophages	ARG1 inhibition, disrupts immunosuppressive metabolite production	Disrupts immunosuppressive phenotype	^87,185^
Epacadostat	Indoleamine 2,3-dioxygenase (IDO) inhibitor	MDSCs	Inhibits IDO, prevents tryptophan depletion	Disrupts immunosuppressive metabolite production	^186^
BPTES	GLS inhibitor	Macrophages	GLS inhibition, blocks glutamine-dependent succinate accumulation	Impairs tumor-promoting pathways, affects M1-like cells	^185^
CAT2 transporter inhibitors	CAT2 inhibitor	MDSCs	Blocks CAT2, combined with L-arginine supplementation	Reverses MDSC-mediated immunosuppression	^188^
DFMO	Ornithine decarboxylase (ODC) inhibitor	Macrophages	ODC blockade, reduces polyamine synthesis	Reduces tumor burden, lessens colon cancer exacerbation	^189^
JHU083	Glutamine antagonist	MDSCs	Restrains expansion, suppresses IDO/kynurenine signaling	Enhances immunotherapy response	^44^

In lipid metabolism, FATP2 acts as a key regulator in MDSCs and TANs by promoting immunosuppressive activity and PGE2 synthesis, and its inhibition attenuates these suppressive functions while slowing tumor progression^103^. TAMs frequently exhibit FAO-driven and lipid-driven M2-like phenotypes supported by tumor-derived lactate that stabilizes HIF-2α via suppression of ATP6V0d2 to promote VEGF expression and angiogenesis^177^. The S100A4–PPARγ–FAO axis further reinforces this lipid metabolism program, and disrupting this pathway reprograms TAMs toward antitumor states^178^. MDSCs rely on FAO for their immunosuppressive function, and inhibition of FAO with agents such as etomoxir reduces oxygen consumption and the expression of suppressive mediators^120^. This intervention restores T cell proliferation and enhances the efficacy of immunotherapy^120^. FAO inhibition also enhances DC activation^115^, whereas FASN-dependent lipogenesis sustains the function of intratumoral regulatory T cells^179^. Broader lipid metabolism modulators like COX inhibitors such as aspirin or celecoxib influence both DCs and MDSCs^103,180,181^. Additionally, blockade of A2AR signaling relieves myeloid cell mediated immunosuppression and enhances antitumor immune responses ^182,183^. Beyond direct metabolic intervention, CD33-targeted therapy with gemtuzumab may provide an additional strategy to deplete suppressive myeloid populations^184^.

Amino acid metabolism represents a critical regulatory layer in TAMs and MDSCs by shaping their immunosuppressive phenotypes. Targeting key enzymes such as ARG1 with inhibitors such as CB-1158 in TAMs^87,185^, and IDO1 with compounds such as epacadostat to prevent tryptophan depletion by MDSCs, disrupts the production of immunosuppressive metabolites^186^. Glutaminase inhibitors including BPTES and CB-839 further impair tumor-promoting pathways^185^. BPTES particularly affects M1-like cells by blocking glutamine-dependent succinate accumulation that is crucial for IL-1β signaling^187^. Moreover, combining L-arginine supplementation with CAT2 transporter inhibition effectively reverses MDSC-mediated immunosuppression^188^. TAM-specific amino acid pathways also influence tumor progression. Ornithine decarboxylase-mediated polyamine synthesis exacerbates colon cancer and its blockade by DFMO reduces tumor burden^189^. Glutamine antagonists such as JHU083 target tumor cells and MDSCs to restrain expansion, suppress IDO1 and kynurenine signaling, and enhance the immunotherapy response^44^.

### Epigenetic drugs to reprogram immune cells

Epigenetic modulators represent a pivotal therapeutic approach for reprogramming the myeloid landscape and reversing tumor-induced immunosuppression (Table 2). HDAC inhibitors targeting the acetyl-CoA/NAD^+^ axis represent a pivotal approach to reprogramming innate immune cells in the TME. Broad-spectrum HDAC inhibitors such as vorinostat, TSA, and panobinostat enhance the pro-inflammatory and antitumor functions of innate immune cells^190,191^. Similarly, class IIa HDAC inhibitors such as TMP195 alongside low-dose pan-HDAC blockade reprogram TAMs in breast and colorectal cancers toward an IL-12-producing phenotype to reduce tumor burden and potentiate anti-PD-(L)1 immunotherapy^192^. HDAC inhibition with CG-745 reshapes the tumor immune microenvironment by reducing immunosuppressive myeloid populations including MDSCs and thereby enhancing the therapeutic efficacy of anti-PD-1 immunotherapy^193^. BET bromodomain inhibitors such as JQ1 or I-BET151 complement these effects by blocking BRD4-mediated transcriptional activation of NF-κB and STAT3 pathways^126,191^. This blockade suppresses immunosuppressive signaling in TAMs and promotes MDSC depletion. In DCs, HDAC1 deletion increases histone acetylation at antigen-processing and cytokine loci to enhance IL-12 production and CD8^+^ T cell priming, which improves responses to PD-1 blockade^194^ (Table 2).

**Table 2:** Epigenetic modulators regulating innate immune cell phenotypes in the TME.

Drug	Category	Target cells	Key mechanisms	Core immune effect	Refs.
Vorinostat, TSA, panobinostat	Broad-spectrum HDAC inhibitor	Myeloid cells	Targeting the acetyl/NAD^+^ axis	Enhance pro-inflammatory and antitumor functions	^190,191^
TMP195	Class IIa HDAC inhibitor	Macrophages	Reprogramming toward an IL-12-producing phenotype	Reduce tumor burden and potentiate anti-PD-(L)1 immunotherapy	^192^
CG-745	HDAC inhibitor	Myeloid cells	Reducing immunosuppressive myeloid populations	Enhance therapeutic efficacy of anti-PD-1 immunotherapy	^193^
JQ1, I-BET151	BET bromodomain inhibitor	Macrophages, MDSCs	Blocking BRD4-mediated transcriptional activation of NF-κB and STAT3	Suppress immunosuppressive signaling and promote MDSC depletion	^126,191^
GSK-J4	Histone demethylase inhibitor (targeting KDM6B)	Macrophages, DCs	Targeting KDM6B within the α-KG/succinate axis	Suppress M2-like phenotype, expand CD103^+^ DCs, and enhance IL-12/type I IFN responses	^195^
5-Azacytidine, decitabine, guadecitabine, zebularine	DNMT inhibitor	DCs, NK cells, macrophages	Upregulating co-stimulatory molecules and IFN-γ production	Promote shift toward M1 macrophage phenotype; enhance DC and NK activation	^6,126,196^
Decitabine (low dose)	DNMT inhibitor	MDSCs	Reducing DNMT-dependent hypermethylation	Deplete MDSCs and reverse tumor-induced immune tolerance	^203^
Vitamin C	TET2 activator (pharmacologic)	Myeloid cells	Augmenting 5-hmC levels on antigen processing genes	Enhance cross-priming and checkpoint therapy efficacy	^198^
UNC1999, GSK126	EZH2 inhibitor	NK cells	Inhibiting EZH2 along the one-carbon/SAM axis	Enhance NK cell maturation	^199–201^
DOT1L inhibitors	DOT1L inhibitor	DCs	Inhibiting DOT1L activity	Enhance DC function	^199–201^
PRMT1 inhibitors	PRMT1 inhibitor	Macrophages	Inhibiting PRMT1 activity	Suppress M2-like polarization	^199–201^
LDHA inhibitors	LDHA inhibitor	Macrophages	Inhibiting lactate production and H3K18 lactylation	Indirectly reduce M2-associated lactylation	^205,206^

*DC* dendritic cell, *DNMT* DNA methyltransferase, *HDAC* histone deacetylase, *IFN* interferon, *MDSC* myeloid-derived suppressor cell, *NK* natural killer, *SAM* S-adenosylmethionine, *TME* tumor microenvironment, *a-KG* a-ketoglutarate.

The α-KG/succinate axis, regulated by key dioxygenases including KDMs and TETs, has a critical role in modulating the epigenetic landscape of tumor-infiltrating myeloid cells. Histone demethylase inhibitors such as GSK-J4 target KDM6B to suppress the M2-like pro-tumoral phenotype in TAMs while promoting the expansion of cross-presenting CD103^+^ DCs^195^. This dual effect enhances IL-12 and type I interferon responses and links α-KG-dependent histone demethylases to effective tumor immunity^195^. DNA methylation dynamics are also regulated through this axis as DNMT inhibitors such as 5-azacytidine, decitabine, guadecitabine, and zebularine enhance DC and NK cell activation by upregulating co-stimulatory molecules and IFN-γ production while promoting a shift toward an M1 macrophage phenotype^6,126,196^. Notably, myeloid-specific deletion of TET2 reprograms TAMs into inflammatory effectors, increases CD8^+^ T cell infiltration, and sensitizes tumors to PD-1 blockade^197^. Pharmacological activation of TET2 by vitamin C augments 5-hydroxymethylcytosine levels on antigen-processing genes in antigen-presenting cells to enhance cross-priming and checkpoint therapy efficacy^198^.

HMTs and their antagonists regulate key epigenetic modifications along the one-carbon and SAM metabolic axis. Inhibitors targeting EZH2 such as UNC1999 and GSK126 and DOT1L enhance NK cell maturation and DC function, respectively, whereas PRMT1 inhibition suppresses M2-like polarization in TAMs^199–201^. Notably, EZH2-mediated H3K27me3 controls cysteine metabolism in HCC, which links PRC2 activity to redox balance and illustrates the integration of epigenetic and metabolic regulation in the TME^202^. Targeting RNA methylation regulators including m^6^A writers, readers, and erasers such as METTL3, YTHDF1, ALKBH5, and FTO offers novel control points over TAM and DC activation as well as MDSC infiltration^18^. Moreover, low-dose DNA methyltransferase inhibitors such as decitabine deplete MDSCs and reverse tumor-induced immune tolerance by reducing DNMT-dependent hypermethylation^203^.

Histone lactylation, particularly at H3K18 (H3K18la), has recently emerged as a pivotal metabolic–epigenetic nexus within the TME. Tumor-derived lactate promotes this epigenetic mark in macrophages to reinforce immunosuppressive IL-10^+^ PD-L1^+^ transcriptional programs by silencing genes such as RARγ, which drives tumor progression in glioblastoma and colorectal cancer^149,204^. Targeting lactate production via LDHA inhibition may indirectly reduce this M2-associated lactylation in TAMs, representing a promising therapeutic avenue^205,206^. Furthermore, MPC activity intrinsically regulates DC metabolism because its impairment enhances H3K18la^147^. This modification hinders DC maturation and CD8^+^ T cell priming, whereas restoration of MPC function or inhibition of lactylation reverses these defects and improves immunotherapy efficacy^147^.

### Combination therapies targeting innate immunity in the TME

Synergistic combinations of metabolic or epigenetic agents with established immunotherapies are being extensively explored to overcome the barriers of the TME (Table 3). Metabolic or epigenetic modulators alone rarely achieve durable tumor control, as they are insufficient to fully reprogram the immunosuppressive TME. Consequently, combinatorial strategies integrating these agents with immune checkpoint inhibitors or standard-of-care treatments have been developed to reinvigorate innate immune circuits, particularly in macrophages, and restore effective T cell-mediated antitumor immunity^207^. Metabolic interventions are most frequently paired with PD-1 blockade. For example, EP4 antagonism using MF-766 reprogrammed TAMs toward an M1-like phenotype, reduced granulocytic MDSCs, and enhanced anti-PD-1 efficacy^208^. Similarly, metformin encapsulated in mannose-modified macrophage-derived microparticles redirected M2-like TAMs toward a pro-inflammatory state, decreased intratumoral MDSCs, and improved the durability of anti-PD-1 responses^209^. In a breast cancer model, sequential IL-1β blockade decreased TAM burden while increasing IL-12-producing CD11b^+^ DCs. This metabolic–cytokine axis remodeling enabled complete tumor rejection following PD-1 blockade and emphasized how priming myeloid metabolism can sensitize innate cells to checkpoint therapy^210^ (Table 3).

**Table 3:** Combination therapies targeting innate immune reprogramming in the TME.

Drug	Target cells	Key mechanism	Primary effect (mono)	Synergy therapy	Synergy effect	Ref.
Entinostat (class I HDACi)	MDSCs, T_reg_ cells	Inhibition of MDSC suppressive mediators and modulation of STAT3 acetylation	Reduction of MDSC-mediated immune suppression	Anti-PD-1	Marked tumor suppression in the RENCA model (~83% reduction)	^211^
MF-766 (EP4 antagonist)	Macrophages, MDSCs, NKs, CD8⁺ T cells, cDCs	Blockade of PGE2–EP4-driven myeloid suppression	Myeloid repolarization and improved cytotoxic infiltration	Anti-PD-1	High complete response rate in the CT26 model (58%) with long-term immunity	^208^
Metformin (Met@Man-MPs)	Macrophages	CD206-targeted macrophage reprogramming with AMPK–NF-κB activation and collagen remodeling	Inflammatory macrophage polarization with reduced suppressive features	Anti-PD-1	High tumor-free rate in the H22 model (60%) with durable immune memory	^209^
Anti-IL-1β Ab	IL-1β-driven monocyte/macrophage lineage	IL-1β neutralization and disruption of inflammatory myeloid support	Reduced macrophage-driven suppression and enhanced IL-12-associated immunity	Anti-PD-1	Near-complete tumor abrogation in the 4T1 model	^210^
CG-745 (pan-HDACi)	CTLs, NKs, macrophages, T_reg_ cells, MDSCs	Pan-HDAC inhibition with histone acetylation maintenance and cytokine induction	Enhanced effector immune activity with reduced suppressive cell populations	Anti-PD-1	Complete tumor clearance in the Hepa1–6 model	^193^
EZH2 inhibitor (EPZ005687)	Macrophages	EZH2 blockade reducing H3K27me3 and inducing macrophage PD-1 expression	Diminished macrophage cytotoxicity	Anti-PD-1	Restoration of macrophage antitumor activity with tumor growth control	^212^

cDC conventional dendritic cell, CTL cytotoxic T lymphocyte, HDAC histone deacetylase, MDSC myeloid-derived suppressor cell, NK natural killer, TME tumor microenvironment, Treg regulatory T, α-KG α-ketoglutarate.

Epigenetic reprogramming offers another promising avenue to reshape the innate compartment for immunotherapy. The class I HDAC inhibitor entinostat neutralized the suppressive activity of polymorphonuclear and monocytic MDSCs, thereby synergizing with PD-1 blockade^211^. Likewise, the pan-HDAC inhibitor CG-745 remodeled the TME by reducing M2-like TAMs, MDSCs, and regulatory T cells while expanding CD8^+^ T and NK cell populations to restore PD-1 responsiveness^193^. By contrast, macrophage-targeted inhibition of EZH2 decreased H3K27me3 but paradoxically elevated PD-1 expression and impaired macrophage cytotoxicity^212^. Anti-PD-1 treatment only partially rescued these effects, suggesting that indiscriminate targeting of the EZH2–H3K27me3 axis can compromise macrophage-mediated immune surveillance^212^. Although direct evidence combining metabolic or epigenetic modulators with standard therapies remains limited, existing findings provide critical design principles. Agents that convert TAMs and MDSCs from suppressive to inflammatory or antigen-presenting phenotypes, such as metformin-loaded microparticles or IL-1β blockade, could be effectively sequenced before PD-1 blockade to enhance antigen release and DC priming, thereby also facilitating synergy with cytotoxic or cellular therapies^209,210^.

Conversely, the deleterious effects of EZH2 inhibition on macrophage function highlight the necessity of tailoring multimodal regimens to specific metabolic–epigenetic circuits and innate cell subsets guided by integrated functional and epigenomic profiling of TAMs, MDSCs, and DCs^212^.

## Conclusions and future perspectives

The interplay between metabolic reprogramming and epigenetic remodeling has emerged as a major determinant of innate immune cell identity and function within the TME. As discussed in this Review, the TME is not simply a nutrient-deprived scaffold, but a metabolically active ecosystem that continuously reshapes the behavior of infiltrating immune cells. Key metabolites, including acetyl-CoA, α-KG, SAM, and lactate, function not only as metabolic intermediates but also as essential cofactors and substrates for chromatin-modifying enzymes, thereby linking metabolic stress to durable epigenetic states. Through this metabolic–epigenetic axis, TAMs, TANs, MDSCs, and DCs can acquire and maintain immunosuppressive or dysfunctional phenotypes, ultimately limiting effective antitumor immunity. Importantly, emerging mechanisms such as histone lactylation and context-dependent innate immune memory further support the idea that these changes are not merely transient adaptations, but can leave persistent functional imprints within the TME.

Despite substantial progress, important questions remain regarding the spatial and cellular heterogeneity of these mechanisms in vivo. Most current metabolic and epigenetic analyses rely on bulk populations, which can obscure distinct cell states within the same tumor. For example, TAMs located in hypoxic tumor cores are likely exposed to metabolic pressures different from those at invasive margins, potentially resulting in divergent epigenetic landscapes that cannot be resolved by bulk approaches. Addressing this complexity will require integrated single-cell multi-omics approaches, including scRNA-seq, scATAC-seq, and emerging single-cell metabolomic methods, together with spatial transcriptomic analyses. These technologies will help define how local metabolic niches shape the plasticity of specific innate immune subsets and may reveal actionable molecular targets that remain hidden in population-averaged datasets.

A major challenge for clinical translation is therapeutic specificity. Because many metabolic enzymes and epigenetic regulators are broadly expressed, systemic inhibition may also impair the function of beneficial immune populations or normal tissues. For instance, strategies that suppress glycolysis in pro-tumor myeloid cells may also interfere with the metabolic requirements of effector lymphocytes. Future therapeutic approaches should therefore prioritize more selective interventions, including myeloid-targeted delivery systems and rational combination strategies. In this context, metabolic priming to shift the TME from a suppressive to a more permissive state before checkpoint blockade may represent a particularly promising framework for overcoming resistance to current immunotherapies.

Overall, understanding metabolic–epigenetic crosstalk provides an important conceptual framework for cancer immunotherapy by linking environmental stress in the TME to stable innate immune dysfunction. Continued integration of metabolism, epigenetics, and immunology will be essential for identifying the regulatory nodes that govern these cell states and for developing more precise therapeutic strategies to restore durable antitumor immunity.
